# Leadership development in genetic counseling graduate programs

**DOI:** 10.1002/jgc4.1906

**Published:** 2024-04-24

**Authors:** Amanda Polanski, April Hall, Catherine Reiser, Katherine Uttal, Ashley Kuhl

**Affiliations:** ^1^ Master of Genetic Counselor Studies Program University of Wisconsin—Madison Madison Wisconsin USA; ^2^ Center for Human Genomics and Precision Medicine, UW Health Madison Wisconsin USA; ^3^ Emeritus Department of Pediatrics University of Wisconsin—Madison Madison Wisconsin USA; ^4^ Department of Pediatrics University of Wisconsin—Madison Madison Wisconsin USA

**Keywords:** education, genetic counseling, leadership, professional development

## Abstract

Leadership is emerging as an important component of health professional training. This study aimed to characterize current leadership development in accredited genetic counseling programs. Semi‐structured interviews with program leadership were conducted to explore their program's leadership curricula and their perspectives on the meaning of leadership and its place in genetic counseling training. Eleven interviews were conducted and focused on seven categories related to study goals. Using the Framework Method, themes were generated within the predefined categories. **Categories** and *themes* included **Defining Leadership** (*Positional* vs *Non‐positional, Beliefs about Leadership, Role of Leadership in the Field of Genetic Counseling*), **Leadership Curricula Origin and Delivery** (*Course‐based and Longitudinal, Explicit vs. Implicit, Origin of Material*), **Role of Faculty and Students** (*Role of Faculty, Expectations for Students and Qualities of Students*), **Skills**, **Evaluation**, **Priority** (*Potential for Improvement, Barriers and Facilitators*), and **Standards** (*Current Incorporation, Potential Incorporation*). All programs had some form of leadership development, but many participants lacked a personal or program definition of leadership. Leadership development varied in curricula and delivery, but most were longitudinal and faculty‐driven, with communication, teaching, advocacy, and collaboration as commonly taught skills. However, leadership development opportunities were rarely labeled as such, and participants identified labeling current leadership development as the top area for improvement. Labeling leadership development could improve assessment of current efforts and the ability to address gaps in leadership curricula. This would lay the foundation for necessary intentional leadership development, in turn helping us better advocate for our patients and the profession.

**What is known about this topic:**

Leadership development has been identified as a gap in global professional health education leading to requirements in many health professional programs. No studies into the incorporation of leadership development in accredited genetic counseling programs have been conducted.

**What this paper adds to the topic:**

This study provides a beginning characterization of the forms that leadership development takes in genetic counseling training programs. It also explores the meaning of leadership in the field and has the potential to inform future research and discussions on leadership development in genetic counseling.

## 1 INTRODUCTION

Leadership, in all levels of clinical care, has been recognized as critical for nurturing high‐quality care cultures (West et al., 2014). To be effective providers and patient advocates, genetic counselors must master the necessary skills to collaborate with other healthcare providers and lead within a clinical environment and the larger healthcare system. However, the meaning of leadership in the field of genetic counseling and its role in genetic counseling training is unclear.

### 1.1 Meaning of leadership

A group's meaning of leadership refers to their specific understanding of leadership, assumptions, and patterns of thinking (Alvesson & Jonsson, 2018). This is different than a definition of leadership which focuses on how leadership is described. Historically, leadership has been framed as leader‐centered, with a focus on individuals in positions of power or influence. From this positional leadership perspective, individuals with certain traits or qualities influencing others to reach organizational goals is the basis of effective leadership (Grys, 2006). However, trends in critical leadership theory have shifted toward a processual leadership meaning that emphasizes ongoing social interaction among all organizational actors (Tourish, 2014). This meaning focuses not on the influence of individuals but rather frames leadership as an ongoing process involving all members negotiating for and determining agency. Despite this shift, society continues to hold onto the image of formal, “heroic” leaders, particularly in the business setting (Alvesson & Jonsson, 2018; Schweiger et al., 2020). The meaning of leadership can be fluid and shaped by individuals' experiences and values. As healthcare providers exhibit leadership as part of their daily duties—collaborating and encouraging contributions with other providers, gathering feedback from patients to develop plans to improve future care, acknowledging mistakes and treating them as learning opportunities (NHS Leadership Academy, 2012)—leadership is taking on new meaning in the healthcare field.

### 1.2 Leadership in healthcare

Healthcare providers must adapt to an evolving and complex system with clinical leaders putting patients first by considering both patient and provider perspectives (Oates, 2012). Clinical leadership has shifted from a focus on provider autonomy to a team‐oriented approach to patient care. An effective clinical leader balances autonomy and accountability to improve patient outcomes (Kushner & Strasser, 2020). Effective leadership improves team performance and patient outcomes in a variety of healthcare settings, including complex care (Akbiyik et al., 2020; Kuluski et al., 2021; Kushner & Strasser, 2020; Mäkinen et al., 2007). There is a lack of consensus on the skills or characteristics required to be considered a leader in healthcare. Examples of skills described in nursing and medical education literature include teamwork, communication, and time management (Stanley & Stanley, 2018; Varkey et al., 2009). Some leadership skills can be difficult to separate from clinical skills used daily, and indeed, many leadership skills are built into routine clinical practice. A compelling example of this is the NHS Medical Leadership Competency Framework (MLCF) which “describes the leadership competences that doctors need to become more actively involved in the planning, delivery and transformation of health services.” Leadership competencies are broken into five domains: “Demonstrating Personal Qualities,” “Working with Others,” “Managing Services,” “Improving Services,” and “Setting Direction” (NHS Leadership Academy, 2012). These competencies are undertaken by a provider as an individual and as part of a team to better the planning, delivery, and transformation of health services.

Genetic counselors are similarly involved in delivering and bettering genetic healthcare. However, there is no such existing leadership framework for genetic counselors. Leadership is less explicitly named but still relevant for genetic counseling patient care as highlighted in the Accreditation Counsel for Genetic Counseling (ACGC) Practice‐Based Competencies. An example is Practice‐Based Competency 22, which emphasizes the need for genetic counselors to “Establish and maintain professional interdisciplinary relationships in both team and one‐on‐one settings and recognize one's role in the larger healthcare system” (Accreditation Counsel for Genetic Counseling [ACGC], 2019a). This competency, for example, would overlap with competencies in the “Working with Others” domain of the MLCF and highlights leadership in multiple settings. The Canadian Association of Genetic Counselors (CAGC) Practice‐Based Competencies for Canadian Genetic Counselors also includes a section on “Leadership” under the Professionalism and Ethical Practice heading (Ferrier et al., 2013). This section includes competencies such as “Act as a resource person, educator, advocate and/or mentor for students, health care professionals and the community” and “Recognize evolving arenas in medical genomics and health care in order to identify the potential for growth within the profession as well as for integration of genetic counsellor practice into new roles.” These competencies further emphasize the importance of leadership in the field of genetic counseling and its continued growth. In addition to their patient care and in line with the above CAGC leadership competencies, many genetic counselors, including recent graduates, regularly take on a leadership role as supervisors of genetic counseling students. 43% of genetic counselors in direct patient care positions report supervision of students as a significant role within their current position (NSGC Professional Status Survey, 2022). Supervision of genetic counseling students has been found to serve at least two primary purposes: promote professional development of students and ensure a minimum quality of care provided (McCarthy & LeRoy, 1998). Supervision represents processual leadership as supervisors both educate and guide students while reflecting on their own professional development and provision of care. Similarly, student supervisees are learning how to receive and implement feedback in their chosen professional field while building necessary clinical skills. Genetic counselors also commonly take on faculty positions. One‐quarter (25%) of genetic counselors who responded to the 2022 PSS held faculty appointments. The most common faculty appointment was Instructor (45%), followed by Assistant Professor (26%). As instructors, genetic counselors demonstrate leadership through teaching, presenting, and time management of lessons and their overall commitments. With the addition of newly accredited genetic counseling programs, the need for leadership in the form of supervisors and instructors continues to grow.

### 1.3 Leadership in healthcare professions education

Along with the changing role of healthcare providers in practice, there has been a call for reform of medical education. In 2010, the Lancet Commission on Medical Education identified gaps and inequities in global professional health education (Frenk et al., 2010). The Commission emphasized the need for health professional training programs to adopt transformative learning to develop leadership and interprofessional education. In response, many health professional training programs have started to incorporate leadership training into their curricula (Lyons et al., 2018; Ross et al., 2021). Table 1 outlines the accreditation standards or entry‐level competencies for many health professions that require leadership development. Common themes across health professions include building and working with a team and using leadership skills to enhance patient care. Multiple accreditation standards and competencies also highlight the leadership skill of advocacy given an evolving healthcare field (Criteria & Procedures for Schools of Public Health & Public Health Programs, 2021; *Domains: Domain 10 Personal, Professional, and Leadership Development*, 2021; Standards and Required Elements for Accreditation of Physical Therapist Education Programs, 2020).

**TABLE 1:** Leadership development requirements for health professional training programs.

Profession	Leadership development requirement	Where
MD (United States)	Yes^a^	Association of American Medical Colleges—Physician Competency Reference Set
MD (United Kingdom)	Yes^b^	General Medical Council—Outcomes for graduates 2018
Nursing	Yes^c^	American Association of Colleges of Nursing—The Essentials: Core Competencies for Professional Nursing Education
MPH	Yes^d^	Council on Education for Public Health 2021 Accreditation Criteria
PT	Yes^e^	Commission on Accreditation in Physical Therapy Education—PT Standards and Required Elements
OT	Yes^f^	Accreditation Council for Occupational Therapy Education—Standards and Interpretive Guide 2018
PA	Yes^g^	American Academy of Physician Associates—Competencies for the Physician Assistant Profession 2021
PharmD	Yes^h^	Center for the Advancement of Pharmacy Education—Educational Outcomes
Audiology	No	
SLP	No	

*Sources*: ^a^Englander et al. (2013); ^b^General Medical Council (2009); ^c^ *Domains: Domain 10 Personal, Professional, and Leadership Development* (2021); ^d^Criteria and Procedures for Schools of Public Health and Public Health Programs (2021); ^e^Standards and Required Elements for Accreditation of Physical Therapist Education Programs (2020); ^f^2018 Accreditation Council for Occupational Therapy Education [ACOTE] Standards and Interpretive Guide (2022); ^g^Competencies for the Physician Assistant Profession (2021); ^h^Medina et al. (2013).

### 1.4 Leadership development in genetic counseling programs

Currently, there is no explicit standard or accreditation requirement for genetic counseling training programs to provide leadership development for students. Despite the lack of formal requirement, accredited genetic counseling programs have demonstrated their intent to shape trainees as leaders. This intent is reflected in program vision and mission statements as well as program objectives. From a review of program websites, phrases about leadership and cultivating leaders in the field were common. Some examples include “cultivate exceptional genetic counseling leaders,” “lifelong learners and leaders in the field,” “promote the field of genetic counseling through clinical care, teaching, research, advocacy, and leadership,” and “To create leaders in genetic‐related healthcare […].”

While reviews of leadership training in other healthcare educational programs like medical (Neeley et al., 2017) and nursing (Morrow, 2015) schools have been conducted, little is known about the state of leadership training in genetic counseling programs. The purpose of this study was to characterize the current state of genetic counseling graduate student leadership development and better delineate the meaning of leadership in the field of genetic counseling. Recognizing the potential for leadership in genetic counseling may allow for future focus on growth of genetic counselors as leaders.

## 2 METHODS

### 2.1 Participants and recruitment

This study included program leadership members from ACGC‐accredited programs in the United States and Canada. A purposive sampling method was used to recruit participants. The Association of Genetic Counseling Program Directors (AGCPD) email listserv was used to invite one representative from each program to participate. Four separate emails were sent, each containing information about the study's goals and requirements, as well as a link to a pre‐interview survey.

### 2.2 Procedures

An interview guide was developed in line with the research aim of this study and drew from existing research on leadership development in healthcare professions (Jefferies et al., 2016; Omar et al., 2020; Smith et al., 2016). Prior to study recruitment, the guide was piloted by members of the research team to ascertain its usefulness in addressing study goals. The pre‐interview survey and sign‐up process were trialed by multiple research team members. The study was deemed exempt by the University of Wisconsin Madison Institutional Review Board (IRB) (IRB#2023‐1188), and informed consent was gathered from each participant.

The study began with a pre‐interview survey to collect information on program and personal demographics. The survey also included questions related to participants' experiences with formal leadership training, as well as their personal and program definitions of leadership. The survey ensured that only one representative per program was participating in the study. Once the survey was completed, participants were directed to an online sign‐up page where they could schedule a 45‐minute interview time slot. After signing up, participants were contacted by a member of the research team via email to confirm their interview time and to receive an overview of the study and interview questions.

The study was conducted from October to December 2022, and 13 individuals completed the pre‐interview survey, while 11 of them underwent a virtual video interview conducted by the primary author. During interviews, the interviewer took field notes of ideas that arose in each interview. Notes focused on highlighting unique points and categorizing general themes in each interview to provide a starting point for future analysis. A semi‐structured interview guide was used to ensure key categories were discussed with each participant while allowing for natural progression and variation based on the points raised by individuals. The pre‐determined seven key categories based on study goals were **Defining Leadership**, **Leadership Curricula Origin and Delivery**, **Role of Faculty and Students**, **Skills**, **Evaluation**, **Priority**, and **Standards**. A list of interview questions is available in the Supporting Information. The audio of interviews was recorded and professionally transcribed using TranscriptionHub. Any names mentioned during the interview and all identifiers were removed from transcriptions prior to analysis. The transcripts were not returned to participants for comment.

### 2.3 Data analysis

Data analysis was carried out using the framework method (Gale et al., 2013). Given that this study aims to characterize an area with little existing literature, this framework was selected to provide both organizational structure and fluidity in comparing trends across and within programs. Following data transcription, both independent coders thoroughly reviewed the interview content and field notes taken by the interviewer. An initial codebook was then created by the coding team consisting of the interviewer and an additional research team member. The codebook was created to chart the thematic analysis as it developed, using both a deductive and inductive coding approach. This method allowed for consistency in obtaining answers to specific research questions while also allowing novel patterns and variations to develop and reflect interviewees' unique perspectives.

Following the first round of independent coding, the analytical framework was further developed, and the codebook was adjusted through reconciliation discussion. A second and final round of coding was then conducted and followed by reconciliation discussion to ensure that no further adjustments to the coding were needed. During the second reconciliation meeting, it was decided that no further coding was necessary as codes had been developed to the point where the research team had no variation in content interpretation. The coded material was then analyzed and sorted into a framework matrix by category and codes, with interview quotes included to illustrate each code. Themes were created to better organize and analyze the different ideas discussed in each category. Finally, the coding team reviewed the analyzed data to ensure agreement on the developed categories and themes.

Notably, multiple members of the research team, including the primary author, have received formal training in leadership theory and practice. While the authors are proponents of leadership training in genetic counseling graduate programs, it was not the goal of the study to prove that leadership curricula and activities are necessary to genetic counseling. Rather, this study was conducted to characterize the current state of student leadership development in genetic counseling graduate programs. Nonetheless, the qualitative analysis carried out for this study was subject to potential bias of the research team. To strengthen analysis objectivity, a second coder without any background in leadership development or involvement in the conception of this study was chosen. All analyses were carried out with the goal of accurately reflecting the current state of genetic counseling graduate programs leadership development.

## 3 RESULTS

### 3.1 Demographics

The survey responses were provided by representatives of 13 out of 57 genetic counseling programs, and due to the small sample size, no demographic details were presented in a table to maintain anonymity. Among the 13 program leadership members who completed the pre‐interview survey, 12 were Program Directors, and their tenure in their current position ranged from under a year to over 20 years. Ten out of the 13 respondents reported having received formal leadership training. Two had not received such training, and one was unsure. The respondents who received formal leadership training attended programs offered by the National Society of Genetic Counselors (NSGC) and the American Board of Genetic Counseling (ABGC), took courses and workshops through their institutions, and received leadership training as part of their non‐genetic counseling graduate education.

### 3.2 Interview categories

Seven categories were explored, and analysis identified several themes within some categories. Categories and their associated themes are listed in Table 2. Verbatim, representative quotations are provided to illustrate categories and themes.

**TABLE 2:** Leadership development topics and themes.

Category	Theme	Definition
Defining Leadership	Positional vs. Non‐positional	Positional: Described leadership in designated roles or jobs Non‐positional: Described the broad nature of leadership and its relevance outside of a formal position
	Beliefs about Leadership	Discussed fundamentals beliefs about leadership and/or individuals capacity for leadership
	Role of Leadership in the Field of Genetic Counseling	Discussed leadership in the genetic counseling field outside of the graduate student experience, including leadership roles like in professional organizations
Leadership Curricula Origin and Delivery	Course‐based and Longitudinal	Course‐based: Discussed leadership delivery delivered in a course setting Longitudinal: Discussed leadership development delivery outside of a single instance in a formal course
	Explicit vs. Implicit	Explicit: Described how leadership curricula and development is labeled and discussed as leadership material Implicit: Described how leadership curricula and development is present but not labeled and discussed as leadership material
	Origin of Materials	Discussed how leadership material was created and developed
Role of Faculty and Students	Role of Faculty	Discussed the role of faculty in student leadership development
	Expectations for Students and Qualities of Students	Expectations: Discussed how, if at all, leadership development and leadership skills are an expected outcome for students Qualities: Discusses the qualities of genetic counseling students in reference to leadership capabilities outside of as a direct outcome of their program's training
Skills		Discussed leadership skills hopefully developed by students throughout training
Evaluation		Discussed how effectiveness of leadership development delivered at their program was evaluated
Priority	Potential for Improvement	Described areas for improvement to leadership development in genetic counseling training, both in their programs and broadly
	Barriers and Facilitators	Discussed barriers and facilitators to genetic counseling programs' incorporation of leadership development
Standards	Current Incorporation	Described if and how leadership development is currently present in ACGC standards
	Potential Incorporation	Described if and how leadership development could be incorporated into ACGC standards

#### 3.2.1 Category 1: Defining leadership

The pre‐interview survey asked each respondent to provide their program's definition of leadership as well as their personal definition. Of the 13 respondents, eight reported that their program currently did not have a definition of leadership, while one was unsure. Two provided learning objectives for their leadership training but did not provide a formal definition. The remaining two provided a definition that guides their leadership training. The common components of program definitions and learning objectives included self‐awareness and understanding, collaboration, and the identification of needs to set a vision.

Leadership includes developing a vision and a mission with input from key stakeholders. Leadership includes exploring ways to innovate and to engage with team members to define and describe what is expected.

Ten respondents provided a personal definition of leadership. One respondent was unsure, and two respondents stated they do not currently have a personal definition of leadership. Common components of personal definitions included teamwork, influencing and empowering others, listening to and respecting other perspectives, and recognizing strengths.

The process whereby an individual or group of individuals work together to develop strategic planning initiatives and goals. One task of a leader is to mentor, delegate, and guide the people that they are leading. Leadership includes listening to dissenting opinions and making difficult decisions, when necessary. It also includes taking responsibility and acknowledging the efforts of others while also giving others a voice.

The process of cooperation with, and inspiration, facilitation and supervision of others to work toward a common goal.

During the interview, participants were not provided with a definition of leadership to avoid influencing their responses. Unprompted, two interviewees commented on the difficulty of defining leadership.

I could define it a million and one different ways than maybe what you're defining it. So I kind of wonder what… How do programs even define leadership right?

##### Theme 1: Positional versus non‐positional

Interviewees spoke about leadership from both a non‐positional and positional lens. Those who focused on a non‐positional lens noted that in their programs, leadership is viewed and taught from a broad perspective. When leadership was discussed from a positional view, leadership roles in organizations were highlighted. Several interviewees noted they discuss the differences between leaders and managers with students and stressed the positional nature of managers as opposed to leaders.

I really wanted students to come away with a broad definition of leadership and that there are many ways to be a leader, many of which don't include some kind of formal role or title or recognition.

##### Theme 2: Beliefs about leadership

Certain beliefs about leadership and individuals' capacity for leadership were noted. Interviewees expressed that leadership skills are foundational and that all people have the capacity to be leaders, although this was not explicitly asked. On the other hand, some interviewees believed that not all individuals have to be or want to be leaders. The interviewees who spoke of the foundational nature of leadership used a non‐positional meaning of leadership throughout the interview. In contrast, those who stated that not all individuals have to be leaders more frequently used a positional meaning, emphasizing the idea of leadership as a position.

Sometimes people think that leaders have to have like these outstanding skills and only certain people can be leaders. And that's so not true. Also, to recognize that each person has the capability of being a leader if they want that type of role and so helping that, encouraging that, it really is for everyone if they want it.

It doesn't mean that everyone has to be a leader. It doesn't work like that. […] You can't be a leader if you don't have followers, right?

Some participants also connected leadership to efforts of diversity, equity, and inclusion (DEI). Specifically, they discussed how positional leadership experiences prior to graduate school are less accessible to societally underrepresented groups and individuals of lower socioeconomic status. Graduate programs were cited as a potential area where equity in leadership training and experiences could be protected.

It's not equal. It's not an equal playing field. Men are more likely, we know that, than women. […] Individuals from marginalized populations have less opportunity […].

[We can] make sure that everybody has good access to leadership training and it's equitable and that everybody is getting an opportunity for those leadership positions.

##### Theme 3: Role of leadership in the field of genetic counseling

The interviews focused mainly on leadership development in graduate programs, but many participants also shared their opinions on leadership in the field of genetic counseling as a whole. Several interviewees emphasized the importance of genetic counselors as leaders due to the profession's small and growing size. They highlighted the importance of professional advocacy and engagement in the profession as examples of how leadership is important in genetic counseling. Interviewees also noted that practicing genetic counselors have leadership skills acquired from their professional counseling experience and graduate training that can be applied to these pursuits. Skills acquired included organization, advocacy for patients and oneself, collaboration, and communication.

It's important that genetic counselors take up leadership roles in the field, because there aren't very many of us. And so somebody needs to do those things.

We recognize especially that genetic counselors are going to have a bigger voice if they take those leadership positions at their institutions, for instance, on advisory committees, whatever it may be. And if we want to keep pushing the profession forward, then we have to be willing to use that voice in a positive way.

Interviewees spoke about the lack of a natural pipeline for formal leadership positions including for genetic counseling program directors. They identified a need for leadership pipelines for practicing genetic counselors and faculty.

And definitely, in other professions like nursing, there are leadership pipelines and just getting genetic counseling students to think about moving up those pipelines and genetic counselors as well.

Participants were split on when leadership training should be delivered. Some believed this process should include leadership training during graduate school; while others stated the place for more robust leadership training was postgraduate and should be elective for those who are interested.

Leadership development, I really see it as a post MS. The biggest area for that development I think personally should be postgraduate school with maybe an introduction in graduate school but all the pursuing of that dependent on individual interest, in being a leader, being in leadership.

#### 3.2.2 Category 2: Leadership curricula origin and delivery

Interviewees were asked questions about how leadership development is incorporated into their programs and how that content was developed. All programs have some form of leadership development present, but the format and origin of that material varied.

##### Theme 1: Course‐based and longitudinal

All participants described some form of leadership training within their courses, including formal lectures, introductions to professional organizations, and seminars on teaching and supervision, and reflections on past and future leadership opportunities. Leadership content was often integrated into courses related to professional issues and development.

More commonly, interviewees described a longitudinal component to their leadership development, which aligned with their program's underlying philosophy or vision. Longitudinal leadership development was defined as leadership development delivery outside of a single instance in a formal course (Table 2). Examples of longitudinal components included expectations for leadership within research teams, community involvement, peer mentorship, advocacy work, and leadership positions facilitated by the program. Some participants felt there was an argument that every aspect of genetic counseling training could support leadership development.

Leadership is really interwoven throughout the curriculum, and it's more of a mindset, in our perspective, and a responsibility.

I probably could argue that 100% of what they're doing is going to support the development of leadership skills. I'd be hard pressed to find something that I couldn't say was supporting the development of leadership skills.

##### Theme 2: Explicit versus implicit

During analysis and in conversation with interviewees, it became clear that what participants considered leadership development material was not always labeled as such for students or in the program. Several respondents indicated they explicitly discussed some aspect of their material as leadership curricula with students or internally within their program.

The pieces that are within the course that I teach, that was very specific. I very intentionally wanted to talk about professional development in terms of leadership and leadership concepts.

The majority of respondents stated that some aspect of their leadership material was not explicitly presented as leadership material, but rather implied leadership curriculum. Of these respondents, some described leadership development as falling under the hidden curriculum—lessons that are essential for student success when learned but not openly taught in classes (Jackson, 1968). Material that was felt to be unlabeled leadership curricula included components of formal lectures, student leadership skills, leadership theories guiding curricula development, and underlying program philosophies. The majority of respondents noted they do not define leadership or explicitly call out leadership development moments for students.

I don't think that [leadership development] is explicitly [incorporated], in fact I know it's not explicitly.

And some of them are a little bit more obvious, and others are a little bit more subtle, especially for the learner. I don't think we call them out like, and this is a moment where you are now learning to become a leader.

##### Theme 3: Origin of material

The interviewees identified four main drivers behind the creation of leadership content in their programs: Faculty, Theory, Outside of Program, and Students. The most mentioned driver was Faculty, which referred to leadership material that originated from the knowledge, experiences, and training of faculty members. Interviewees explained that faculty members' prior experience on professional boards and leadership training and/or courses they or other faculty have attended were the primary sources of leadership material.

Some of that is from personal experience. And other faculty, you know, having done things like a strength finders exercise ourselves thinking this is something that would be a value to the students. So some of those experiences have been drawn in plus my own personal involvement with the Board of Directors for ABGC and NSGC.

Several participants reported their leadership development involved a leadership theory, framework, model, or assessment tool. A list of reported theories and tools can be found in Table 3. The CliftonStrengths Assessment (Rath, 2007)—formerly CliftonStrengths Finder—was the most frequently used tool.

**TABLE 3:** Leadership theories, frameworks, models, and assessment tools used in genetic counseling programs.

Leadership theory
CliftonStrengths Assessment^a^
Transactional vs. Transformational^b^
Authentic Leadership^c^
Lewin's Leadership Theory^d^
Hersey–Blanchard Situational Leadership^e^
The 7 Habits of Highly Effective People^f^
DiSC Assessment^g^
The Leadership Framework Self‐Assessment Tool^h^
Servant Leadership Model^i^

*Sources*: ^a^Rath (2007); ^b^Bass and Riggio (2006); ^c^George (2003); ^4^Lewin (1997); ^e^Hersey and Blanchard (1977); ^f^Covey (1991); ^g^John Wiley, and Sons, Inc (2013); ^h^NHS Leadership Academy (2012); ^i^Wheeler (2012).

Several participants stated there is an outside body which has provided leadership development material to the program and/or students. Participants also described opportunities for students to participate in a health professionals leadership training program. One of these described student involvement opportunities was participation in a Leadership Education in Neurodevelopmental and Related Disabilities (LEND) program (Association of University Centers on Disabilities [AUCD], 2011). Another source of leadership development material was the Leadership Workshop for Genetic Counseling Students created and facilitated by Elizabeth Kearney, MS, CGC, MBA from 2009 to 2019.

Students' interests, actions, and experiences influenced the leadership material and opportunities available to them. Participants stated leadership opportunities were self‐selected by students or that student interest and requests influenced their leadership material. Interviewees noted that empowering students and allowing them to follow their own passions resulted in students taking on roles of leadership.

Some of this may be driven by the students or the individual student. If, you know, someone wants to self‐nominate for president, they've sort of demonstrated a desire to do some things on the leadership side […].

#### 3.2.3 Category 3: Role of faculty and students

While discussing the incorporation of leadership development in their programs, interviewees highlighted the roles of faculty and students. Participants were asked questions about faculty role in leadership curricula development and goals for students.

##### Theme 1: Role of faculty

Upon analysis, three main roles of faculty were identified when it came to student leadership development—Modeling, Facilitation, and Mentorship. Participants discussed how modeling leadership skills and opportunities is an important role of faculty. This included program leadership as well as clinical supervisors and other faculty. Interviewees noted this was an indirect way to encourage students to learn and practice leadership.

I try to be a role model for […] how their professional identity can include leadership.

Facilitation was also discussed by interviewees. They shared methods of facilitating leadership opportunities and development for students outside of the structured program. This often included passing along information about positions on committees or in organizations.

Mentorship was described as important for faculty to mentor students both in and out of the program in addition to the peer mentoring that often occurs in programs between first‐ and second‐year students. Participants explained how their own careers have been shaped by mentorship as evidence of its importance.

We really work to identify opportunities for our learners and think of ways that we can help our students.

Part of […] making individuals become effective leaders is mentorship. And so that's mentorship both within the program and then after, because I think sometimes it takes that extra push of a mentor to get people to recognize their capacity for leadership.

##### Theme 2: Expectations for students and qualities of students

All interviewees expressed some expectation for students gaining leadership skills from their genetic counseling training. Participants emphasized the importance of developing introductory leadership skills and recognizing future leadership opportunities. This included the reflection that the purpose of graduate school is to produce entry‐level genetic counselors and that leadership should not be an expectation of new graduates.

Not all my graduates are going to be like the director or the person who's at the table always making the last decision, but I would want all of my graduates to have leadership skills at some capacity.

I don't think […] a student has to leave a program being an excellent leader to be a great genetic counselor.

Unprompted, many participants discussed leadership qualities of genetic counseling students unrelated to their program's training. They noted that students who successfully match to genetic counseling programs tend to have leadership experience or evidence of leadership skills in their application.

We're looking for leadership skills in our applicants, so maybe we're just biased into picking leaders because we recognize the skills needed to be genetic counselor are actually leadership skills […].

#### Category 4: Skills

All participants were asked if there were specific leadership skills they hoped students developed over the course of training. Participants listed leadership skills as summarized in Table 4. The two most reported skills were communication and teaching (4 times). The skill of communication was described in both a patient care and professional team setting. Teaching was deemed important for education of patients, colleagues, and the greater community. Advocacy, collaboration, giving and receiving feedback, organization, and self‐awareness were also frequently reported (3 times). Many of the skills were discussed as inherent skills of genetic counseling, while others were explained in the context of professional development.

I think that genetic counselors' skill set that we're training them to have anyway also lends itself very well to leadership; good communication, being able to talk to people about their goals and come to a mutually agreed upon agenda.

We do have that kind of as a formal checkpoint for those skills, but it's not explicitly called leadership, it's more under the cloak of just overall professional skills.

**TABLE 4:** Leadership skills for genetic counseling students.

Leadership skill	Times discussed
Communication	4
Teaching	
Advocacy	
Collaboration	3
Giving and Receiving Feedback	
Organization	
Self‐awareness	
Public Speaking/Presenting	2
Recognizing Leadership Opportunities	
Seeking Different Perspectives	
Team Building	
Trust	
Coordinating	1
Counseling	
Delegation	
Emotional Intelligence	
Flexibility	
Having Difficult Conversations	
Humility	
Listening	
Ownership	
Planning	
Recognizing the Needs of Others	
Respect	
Setting Agendas	
Supervision	
Time Management	

#### 3.2.4 Category 5: Evaluation

Participants were asked how they assess the effectiveness of the leadership development delivered at their program. None of the participants reported a method of formal evaluation (defined as using a defined metric or framework to assess leadership development). Participants did not report assessing students' self‐perception of their leadership development.

So we're not yet doing a good job of formal evaluating. […] I just haven't done a good search of if there are self‐report instruments or things like that where you can gauge readiness for leadership or interest in leadership or […] scales out there that we could track.

Student feedback through course surveys and conversations with faculty were shared as ways to evaluate leadership development. Course evaluations for specific leadership lectures are also utilized. Check in meetings with faculty and exit interviews for soon‐to‐be graduates were discussed as opportunities for students to reflect on leadership goals and progress.

Probably mostly just by how the students respond to it. Is it something that's interesting to them? Do they look like they're bored? It wasn't necessary. If there's any other things they'd like to know about leadership that we didn't talk about. How they respond when they talk about what leadership opportunities they might consider in the future?

Several participants discussed evaluation through reflection on ACGC Practice‐Based Competencies (PBCs) for students. By viewing PBCs as leadership skills, faculty and students are reflecting on their growth in those areas as part of overall competency.

No, we don't specifically ask [leadership] but yes, in the sense that we give them the practice based competencies and we ask them to holistically think about their training, where they are in their training and what they need to develop.

Participants noted they do not evaluate students as leaders, but many tracked leadership outcomes for alumni of their programs. This included leadership positions, supervision roles, and publications. In programs that survey alumni periodically about gaps in training and leadership has not been identified as an area where alumni are unsatisfied.

We ask about all the different components of the program and are they satisfied with the level of education that they got on those different components. So, leadership is one of those areas and they report being satisfied.

#### 3.2.5 Category 6: Priority

Participants were asked to describe how leadership development was prioritized in their program. Participants were divided with some noting that leadership development was not a priority for their program. Reasoning included the need to fill other curricular gaps and a belief that leadership development should primarily occur postgraduate training.

Yes, to us I don't think it's a priority. I think that's more of an ongoing professional development kind of thing for those who are interested in it.

One participant noted the lack of priority and stated they would like to address that in the future. Several participants explained leadership development was a priority given it was part of their overall mission or naturally built into other components of training. One noted that while students developing leadership skills was a priority, formal lectures on leadership were not.

It is prioritized. All the skills that we're trying to build are important skills for leadership.

##### Theme 1: Potential for improvement

Participants were asked to identify areas for improvement to leadership development in genetic counseling training, both in their programs and broadly. The most common response participants shared was the need to explicitly label leadership development material. Of those who shared this response, several noted it would be important to explain leadership as a professional value and why it is present in genetic counseling training. One participant elaborated further and said a definition for leadership and leadership training objectives was needed for the field of genetic counseling.

If genetic counseling instructors, faculty leadership could verbalize it. [If they] could make it explicit that yes, this is one of our professional values and try to promote and value so that it's not hidden for students who may not catch the hidden curriculum all the time.

Participants reflected on the potential of collaboration with larger networks. This included working with campus resources and other genetic counseling programs to create leadership material and opportunities. Ideas included leadership internships, shadowing opportunities, a leadership camp, creating student governments within programs, and having students join committees or an NSGC Special Interest Group.

So having some part‐time kind of training that might be useful for programs that don't necessarily have the resources to put something together themselves or having a leadership internship […]

The question of timing was also raised. Participants wondered when in training would be most beneficial for leadership material to be delivered and what aspects of leadership development were better delivered to new graduates.

There's potential to add leadership development more formally, but my question becomes, is it reasonable to make it a required or is it something like supplemental or is it a postgraduate certificate?

The importance of investing in leadership more broadly in the field was reiterated. Some participants stated increased investment in program faculty and entry‐level genetic counselor leadership development would have a trickle‐down effect on students.

As program directors, we are leaders. But I don't know how good our leadership skills are. [H]aving us be more trained in leadership so that we cannot only use that to continue to improve our programs and continue to advocate for our programs at our unique institutions, but also so that we could use that for student training.

##### Theme 2: Barriers and facilitators

**FIGURE 1:**
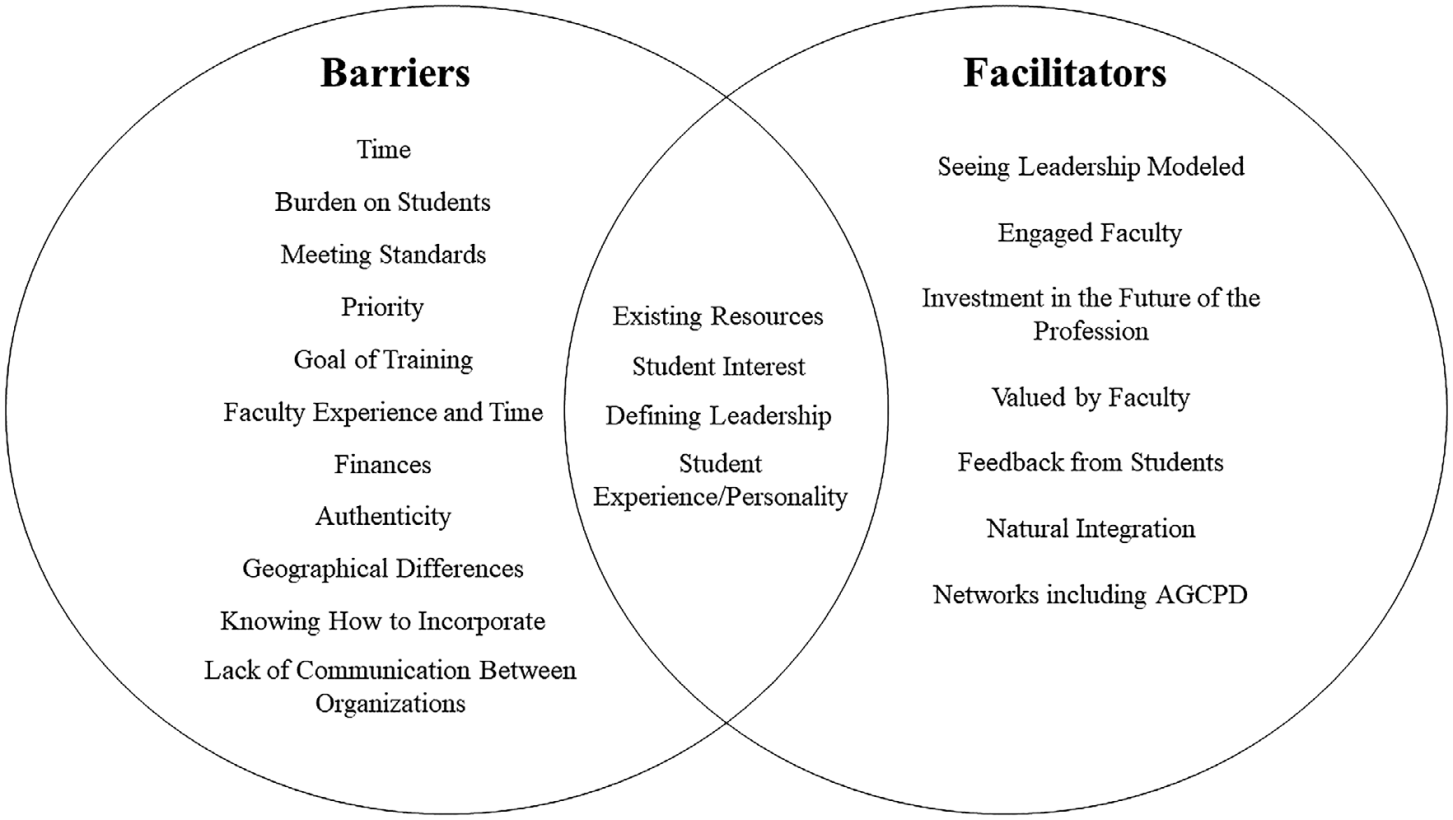
Barriers and facilitators to leadership development incorporation. Barriers and facilitators to leadership development incorporation for genetic counseling graduate programs.

All participants were asked to identify barriers and facilitators to genetic counseling programs' incorporation of leadership development (Figure 1). Overwhelmingly, “Time” was identified as a barrier to incorporation with participants noting the short period of genetic counseling training. In addition to the constraint of time, participants raised a concern for placing a “Burden on Students” by adding this material to an already intense program. “Priority and Meeting Standards” were also discussed, noting the large number of required standards that programs must meet and the need to prioritize those and areas of clinical training over leadership development.

Frequently, participants noted “Seeing Leadership Modeled” as a facilitator to leadership development. Particularly, they noted that having individuals as faculty or clinical supervisors who model leadership allows for students to better understand the importance and potential of leadership development. Similarly, faculty were highlighted as facilitators in other ways. Specifically, “Engaged Faculty” and their “Investment in the Future of the Profession” were discussed as characteristics and values of faculty that could aid incorporation of leadership development.

Some factors were discussed as both barriers and facilitators. The lack or presence of “Existing Resources” for leadership development was seen as a barrier and facilitator, respectively. Interviewees not only identified having structured leadership material for programs to adopt as helpful but also stressed the current absence of these materials as a barrier. “Student Interest” in leadership development was more often seen as a facilitator, while “Student Experience and Personality” was more often seen as a barrier. Participants noted some students' lack of leadership experience or certain personality characteristics may disincline them from desiring leadership development in their training.

#### 3.2.6 Category 7: Standards

All participants were asked if they believed leadership development was already present in ACGC standards. They were also asked if they believed that ACGC standards should include leadership development. The questions related to ACGC standards were provided prior to the interview, but a copy of the current Standards was not given for reference.

##### Theme 1: Current incorporation

Seven participants stated that leadership development was not currently incorporated into ACGC standards. Four participants stated that leadership development was present in ACGC standards. When discussing its presence, three qualified their response by specifying that the word leadership may not be present, but that leadership skills and experiences were included.

When you look at the standards, if you're looking […] for leadership, you can see it, but I'm not so sure that it's written specifically.

##### Theme 2: Potential incorporation

Eight participants stated that leadership development should not be included in ACGC standards. Their reasonings ranged from concern over the prescriptive nature of standards to a belief that leadership development was not a critical component of genetic counseling training. Two also noted it may take away from the individuality of programs. One participant stated that the focus of leadership felt too narrow, and that professional development would be more appropriate.

I don't think it needs to be in the standards specifically, because it doesn't need to be a focus at every program.

Three participants stated leadership development should be included. They elaborated on this stance by stressing the importance of how the standard is written. A standard that both provides guidance and allows programs flexibility with their interpretation was described as ideal.

I do think it should be present […], I'd rather see it as a standard which gives programs some flexibility in terms of how they want to incorporate it.

## 4 DISCUSSION

This study conducted semi‐structured interviews with program leadership to provide an initial characterization of leadership development in accredited genetic counseling programs. We explored seven categories related to leadership development and identified several themes (Table 2). All participants reported some form of leadership development in their program, with varying curricula and delivery methods, largely longitudinal and driven by faculty. Despite these variations, commonly desired leadership skills, barriers, and facilitators to incorporation were identified.

Participants were asked to list leadership skills gained over the course of student training. Some skills were also reflected in program and individual definitions of leadership including self‐awareness and collaboration. This may demonstrate the importance of an underlying definition of leadership to guide the teaching of leadership skills. By reflecting on skills rather than competencies, there is an opportunity to explore how leadership skills overlap with the Practice‐Based Competencies as set by ACGC. The majority of participants stated leadership was not present in the PBCs. Of those who stated it was, the majority noted it was implied rather than explicit. The words “leadership” and “leader” are not present in the ACGC PBCs (ACGC, 2019a). But as participants noted in their own curricula, the lack of explicit label does not mean leadership is not present. Many of the leadership skills most frequently taught in genetic counseling programs, such as communication, teaching, advocacy, self‐awareness, collaboration, and organization, are reflected in the ACGC PBCs (ACGC, 2019a). For example, advocacy is required for PBC 19, “Advocate for individuals, families, communities and the genetic counseling profession” and self‐awareness for PBC 20, “Demonstrate a self‐reflective, evidenced based and current approach to genetic counseling practice.” Despite the lack of explicit mention of leadership in the PBCs, the inclusion of multiple leadership skills identified by participants suggests a link between leadership development and the competencies required of entry‐level genetic counselors. As noted by participants, many skills of genetic counseling are inherently leadership skills. But despite this observation, participants noted multiple barriers to the incorporation of leadership development.

The top barrier to incorporating leadership development was “Time.” Genetic counseling programs vary in length but are required to provide training over a minimum of 21 months or two academic years (Accreditation Counsel for Genetic Counseling [ACGC], 2019b). “Time” is a challenge faced by other health professional training programs including medical school (Jefferies et al., 2016). One solution to this challenge is to identify overlapping areas between leadership development and existing courses and clinical experiences, allowing for the integration of leadership teaching without adding more time to the curriculum. This may include labeling leadership content in formal lectures or providing students with a list of leadership skills they will develop and their connections to ACGC PBCs for reflection. A more intentional approach to current leadership development could help address the issue of limited curricular space and time while still providing students with essential leadership skills.

Only four interviewees mentioned explicitly labeling leadership development within their program. Teaching of skills as described by participants occurs in existing modules, and labeling leadership development explicitly could increase students' awareness of leadership and its importance in clinical care (Jefferies et al., 2016; Webb et al., 2014). Additionally, interviewees recognized the potential for labeling leadership development explicitly and moving away from its place in the hidden curriculum, which is based on unwritten social norms that can exclude marginalized groups. Explicit branding of skill development as leadership development could promote increased accessibility and inclusivity of leadership within the field and allow for natural integration in line with the longitudinal nature of most programs' current leadership development delivery.

### 4.1 Defining leadership

The importance of understanding what leadership means in genetic counseling is highlighted in this study. Many interviewees reported a lack of personal or program definition of leadership, which may have contributed to the absence of labeled leadership development in programs. Without a clear definition of leadership, there are challenges of assessing leadership in genetic counseling programs both formally and informally. For example, several participants note that leadership is informally assessed through student feedback, which may be difficult to interpret if students are not provided with a formal definition of leadership. Assessments are important in tracking the effectiveness of leadership curricula, and without more explicit labeling, further exploration or validation of current efforts will be challenging.

Shared components of leadership definitions in genetic counseling programs were identified, including self‐awareness, collaboration, listening to and respecting other perspectives, and recognizing strengths within a team. Both positional and non‐positional views were discussed. Different views of leadership and its meaning are common across healthcare fields. In both clinical practice and education, work is being done to separate leaders from managers and to define leadership (Elwell & Elikofer, 2015; Oates, 2012). Defining leadership has been recognized as the first step in developing a medical leadership program (Yau et al., 2022) and could aid in ensuring consistency in future research. Therefore, continuing to develop the meaning of leadership in genetic counseling and within programs could maximize the potential of student leadership development and leadership in the field.

### 4.2 Potential of leadership in genetic counseling

The field of genetic counseling is growing and evolving rapidly as genetic counselors enter new healthcare spaces. The milestones for the profession have paralleled the growth and vision of individual genetic counselors' professional development (Baty, 2018). Interviewees noted the importance of developing leadership skills to advocate for the profession and create leadership pipelines for practicing genetic counselors and faculty. Investing in leadership development for all students during graduate training could provide an equitable base of qualified leaders and clarify a pathway for those interested in positional leadership. With the changing healthcare landscape, the need for leadership development of clinicians has been highlighted (Law et al., 2020). Developing leadership skills like those reported in this study could help genetic counselors address opportunities and challenges that arise as they move into new frontiers. Graduate programs providing introductory leadership development could enhance entry‐level genetic counselors' ability to care for and advocate for patients, the profession, and students as they move into supervisory roles (Akbiyik et al., 2020; Kushner & Strasser, 2020; Mäkinen et al., 2007), similar to other professions.

### 4.3 Future directions and limitations

The study found that all interviewed participants reported some form of leadership development in their programs. However, further research is needed to explore students' perception of their leadership development and identity as leaders. This is particularly important in the genetic counseling field, where the meaning of leadership and the effectiveness of current efforts are not yet fully understood. Similar issues have been raised in the medical field in the United Kingdom, where despite a mandate for medical students to graduate with adequate leadership and management skills (General Medical Council, 2009), junior doctors still report feeling unprepared for practice due to lack of leadership knowledge and difficulties with teamwork, communication, and quality improvement skills (Monrouxe et al., 2014). Although the study provides an initial understanding of leadership development in genetic counseling programs, it is important to note that only 11 out of 57 programs were represented in the interviews. More targeted efforts are needed to explore and characterize leadership curricula and opportunities in genetic counseling programs to shape the meaning and potential of leadership in the field.

## 5 CONCLUSION

Provider leadership is critical as healthcare continues to change and challenge. Leadership development in graduate training programs can provide genetic counselors with the skills and experience to navigate these evolving systems and advocate for the place of genetic counselors within them. This study found all surveyed ACGC programs incorporate some form of leadership development. However, there was a lack of consensus in leadership curricula and delivery with few programs explicitly labeling their leadership development. To ensure students develop the desired leadership skills, efforts can be made to call out the existing leadership development lessons and opportunities. While there is limited time for additional curricula to be added, branding current modules as leadership development could help students understand leadership and their own capability. As leadership is critical for the growth of the genetic counseling profession, efforts toward cultivating leaders are an exciting investment in the future and longevity of this field.

## AUTHOR CONTRIBUTIONS

Author A.P. confirms that they had full access to all the data in the study and take responsibility for the integrity of the data and the accuracy of the data analysis. All authors gave final approval of this version to be published and agree to be accountable for all aspects of the work in ensuring that questions related to the accuracy or integrity of any part of the work are appropriately investigated and resolved.

## CONFLICT OF INTEREST STATEMENT

All authors declare that they have no conflict of interest.

## ETHICS STATEMENT

Human studies and Informed consent: This study was reviewed and granted an exemption by the University of Wisconsin—Madison Minimal Risk Research Institutional Review Board. All procedures followed were in accordance with the ethical standards of the responsible committee on human experimentation (institutional and national) and with the Helsinki Declaration of 1975, as revised in 2000. Implied informed consent was obtained for individuals who voluntarily completed the pre‐interview survey and interviews.

Animal studies: No non‐human animal studies were carried out by the authors of this article.

## Supporting information


Data S1.

## Data Availability

The data that support the findings of this study are available from the corresponding author upon reasonable request.
