# Supplementary material for: Leadership development in genetic counseling graduate programs
Source: J Genet Couns. 2024 Apr 24;34(1):e1906. doi: 10.1002/jgc4.1906 (PMC11735175; doi:10.1002/jgc4.1906)
Supplement: Supplementary file 1 — Data S1. [file JGC4-34-0-s001.docx]

Interview Questions

How is leadership training incorporated into your program?

- Is leadership development delivered in a longitudinal manner throughout the program or in stand-alone trainings or courses?
  - If longitudinal, how much total time is dedicated to leadership training?
  - If course based, how many credit hours are the courses?
- What courses in your program incorporate leadership within the content?
- Are there plans to introduce or make changes to your curricula to integrate leadership?
- Does your program have a lead person for leadership development in the curricula?
  - If so, is this a designated position or an informal role?
- Outside of coursework, what other opportunities are there for students to develop leadership in your program?

How was your leadership content developed?

- What guided your leadership content development?
- What, if any, leadership competency models, frameworks, or theories are integrated into your leadership development training?
  - What led you to select that model/framework/theory?

What are the goals of leadership development in your program? How were these determined?

- How do you document goals related to leadership development?
  - Are there specific leadership skills you hope students develop?
- Does your program evaluate students' perception of their leadership development in an exit interview and/or survey?
- Do you track alumni leadership roles as a measure of your program’s training?

How do you assess the effectiveness of your leadership development?

- Have you formally evaluated the leadership development delivered at your program?
  - If so, how?

How have you prioritized leadership development in student training?

- What influences the priority of leadership development for your program?
- Has the priority changed over time?
  - If so, how?
  - What led to this change in priority OR What led to the priority remaining consistent?

How do you think leadership development could be improved in your program? In GC programs as a whole?

- What barriers prevent program incorporation of leadership development- for both your program and programs as a whole?
- What factors facilitate program incorporation of leadership development- for both your program and programs as a whole?

What role should leadership development play in GC programs?

- Do you feel leadership development is already a part of ACGC standards?
- Should there be ACGC accreditation standards related to student leadership development?
- If there were standards, how do you think leadership might be assessed in a program’s curricula?
- What excites you about the potential of leadership development in the training of GC students?

Do you have any thoughts or comments related to leadership and genetic counseling that you haven’t yet had the opportunity to share in this interview?
